# Snake venom galactoside-binding lectins: a structural and functional overview

**DOI:** 10.1186/s40409-015-0038-3

**Published:** 2015-09-24

**Authors:** Marco A. Sartim, Suely V. Sampaio

**Affiliations:** Departamento de Análises Clínicas, Toxicológicas e Bromatológicas, Faculdade de Ciências Farmacêuticas de Ribeirão Preto, Universidade de São Paulo (USP), Avenida do Café, s/n, Ribeirão Preto, SP CEP 14040-903 Brazil

**Keywords:** Snake venom, C-type lectin, Galactoside-binding protein, Carbohydrate recognition domain, Glycoconjugates, Inflammatory response, Antitumor activity, Bactericidal activity, Mitogenic activity, Platelet aggregation

## Abstract

Snake venom galactoside-binding lectins (SVgalLs) comprise a class of toxins capable of recognizing and interacting with terminal galactoside residues of glycans. In the past 35 years, since the first report on the purification of thrombolectin from *Bothrops atrox* snake venom, several SVgalLs from Viperidae and Elapidae snake families have been described, as has progressive improvement in the investigation of structural/functional aspects of these lectins. Moreover, the advances of techniques applied in protein-carbohydrate recognition have provided important approaches in order to screen for possible biological targets. The present review describes the efforts over the past 35 years to elucidate SVgalLs, highlighting their structure and carbohydrate recognition function involved in envenomation pathophysiology and potential biomedical applications.

## Introduction

Lectins are non-enzyme and non-immune proteins capable of binding reversibly, specifically and non-covalently to carbohydrates, and have been isolated from plants, microorganisms and animal sources [1]. Although distinct lectins lack primary structural similarity, they share similar glycan-binding specificities in which the carbohydrate recognition domain (CRD—represented by a segment of limited amino acid residues within the lectin) is responsible for the glycan-interaction activity [2, 3].

Among animal sources, calcium dependent (C-type) lectins have been identified in snake venoms and are classified into two distinct groups: the true C-type glycan-binding lectins; and C-type lectin-like proteins. The C-type lectin-like (also named “snaclecs” from snake venom C-type lectins [4]) are heterodimeric proteins with CRD-related lectin-like domain that are not capable of interacting specifically with sugars [5]. On the other hand, the glycan-binding C-type lectins are homodimeric proteins, composed of two identical disulfide-linked polypeptide monomers with molecular mass of approximately 15 kDa, presenting a functional CRD that binds to carbohydrates and are capable of inducing hemagglutination by recognizing erythrocyte surface glycoconjugates [6].

The first studies on snake venom lectins were reported by Flexner and Noguchi [7] wherein the authors observed agglutination activity of erythrocytes and leukocytes by a variety of venoms. However, the first data on the isolation of a snake venom lectin was reported by Gartner et al. [8] almost 80 years later, describing the purification and characterization of thrombolectin, the first galactoside-binding lectin isolated from *Bothrops atrox*. Since then, several reports on purification and both the structural and functional characterization of snake venom lectins have been described from the snake families Viperidae and Elapidae, including from the genera *Bothrops*, *Crotalus*, *Bitis*, *Agkistrodon*, *Lachesis*, *Dendroaspis* and *Trimeresurus*. Most snake venom glycan-binding lectins are members of the C-type galactoside-binding proteins due to their ability to interact with terminal galactoside residues in a calcium-dependent manner [8–21]. The lectin isolated from *Lachesis muta stenophrys* (LmL) was first classified as lectin-like by the authors [10]. However, taking into account its molecular and functional aspects (which will be further discussed in this review), a more appropriate definition was provided by classifying it as a true galactose-binding lectin.

The present review aims to introduce the efforts over the past 35 years on the study of snake venom galactoside-binding C-type lectins (SVgalLs) by providing insights on the structural and biological activities associated with its glycan recognition pattern.

## Review

### Molecular structure analysis

Important advances in molecular analysis and structure determination of SVgalLs have been performed during the past years. Although the detail of structural information on these lectins varies from a single SDS-PAGE molecular mass analysis to a complete quaternary structure determination, overall molecular aspects indicate a high similarity among these proteins as will be discussed in this section. Currently, rattlesnake lectin (RSL), from *Crotalus atrox* venom, is the only SVgalL whose complete structure was determined by X-ray crystallography [22], and is widely applied in structural analysis comparisons with others lectins from this class [6].

All SVgalLs are homodimeric proteins composed of disulfide-linked monomers presenting molecular mass varying from 14 to 16.2 kDa. The primary structures of 12 SVgalLs were determined, and presented from 134 to 136 amino acid residues as described for the following lectins: RSL—rattlesnake lectin from *Crotalus atrox* [23], ApL—*Agkistrodon piscivorus piscivorus* lectin [15], BaL—*Bitis arietans* lectin [13], CrL—*Crotalus ruber* lectin [18], BiL—*Bothrops insularis* lectin [16], BmLec—*Bothrops moojeni* lectin [24], BpalL—*Bothrops pauloensis* lectin [21], BJcuL—*Bothrops jararacussu* lectin [25], BpirL—*Bothrops pirajai* lectin [17], LmL—*Lachesis muta stenophrys* lectin [26], ToL—*Trimeresurus okinavensis* lectin [27] and TsL—*Trimeresurus stejnegeri* lectin [28]. Amino acid sequence analysis among the referenced SVgalLs using BLAST searching tool [29] showed an identity degree from 82 to 97 % among them, indicating a high primary structural similarity within these lectins.

**Fig. 1:**
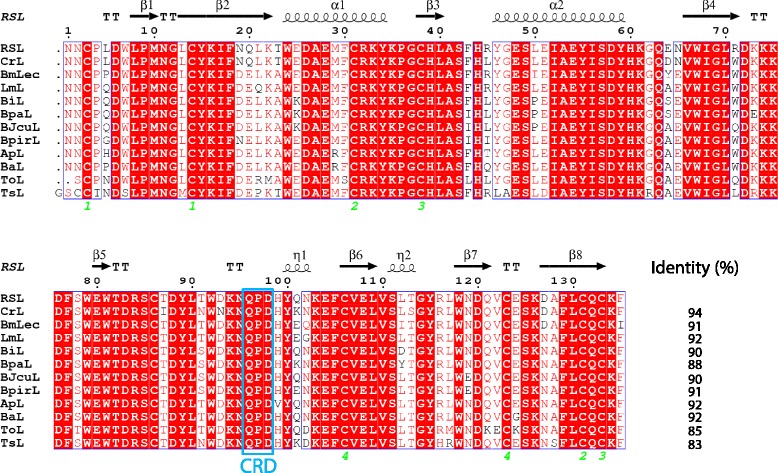
Multiple alignment of SVgalL. The multiple alignment was performed using the program Clustal-X, and sequence similarities using ESPript 3.0 [73] with structure prediction based on RSL structure [PDB: 1JZN]. Fully conserved residues are highlighted in red background. Cystein residues involved in the interchain disulfide link are indicated by green numbers. Carbohydrate recognition domain (CRD) is indicated by the blue box. Primary structure according to lectins: RSL [GI: 126130], ApL [GI: 510120659], BaL [GI: 34922645], CrL [GI: 118572769], BiL [GI: 82126834], BmLec [24], BpalL [GI: 527504051], BJcuL [25], BpirL [GI: 510120660], LmL [GI: 1881829], ToL [27] and TsL [GI: 7674107]

A multiple alignment comparing RSL primary and secondary structures with the mentioned lectins was performed. As shown in Fig. 1, sequence identities vary from 83 to 94 % when compared to RSL, presenting important conserved structural features such as positioning of cysteine residues. The eight to nine highly conserved cysteine residues within these SVgalLs indicate the presence of four intramolecular Cys-Cys disulfide-linkages (Cys^3^–Cys^14^, Cys^31^–Cys^131^, Cys^38^–Cys^133^ and Cys^106^–Cys^123^), as determined for the RSL structure (Fig. 1). Furthermore, Cys^86^ is directly involved in the interchain disulfide link in order to compose the dimeric structural pattern of RSL [22], as observed with the same positioning residue for the other SVgalLs (Fig. 1).

**Fig. 2:**
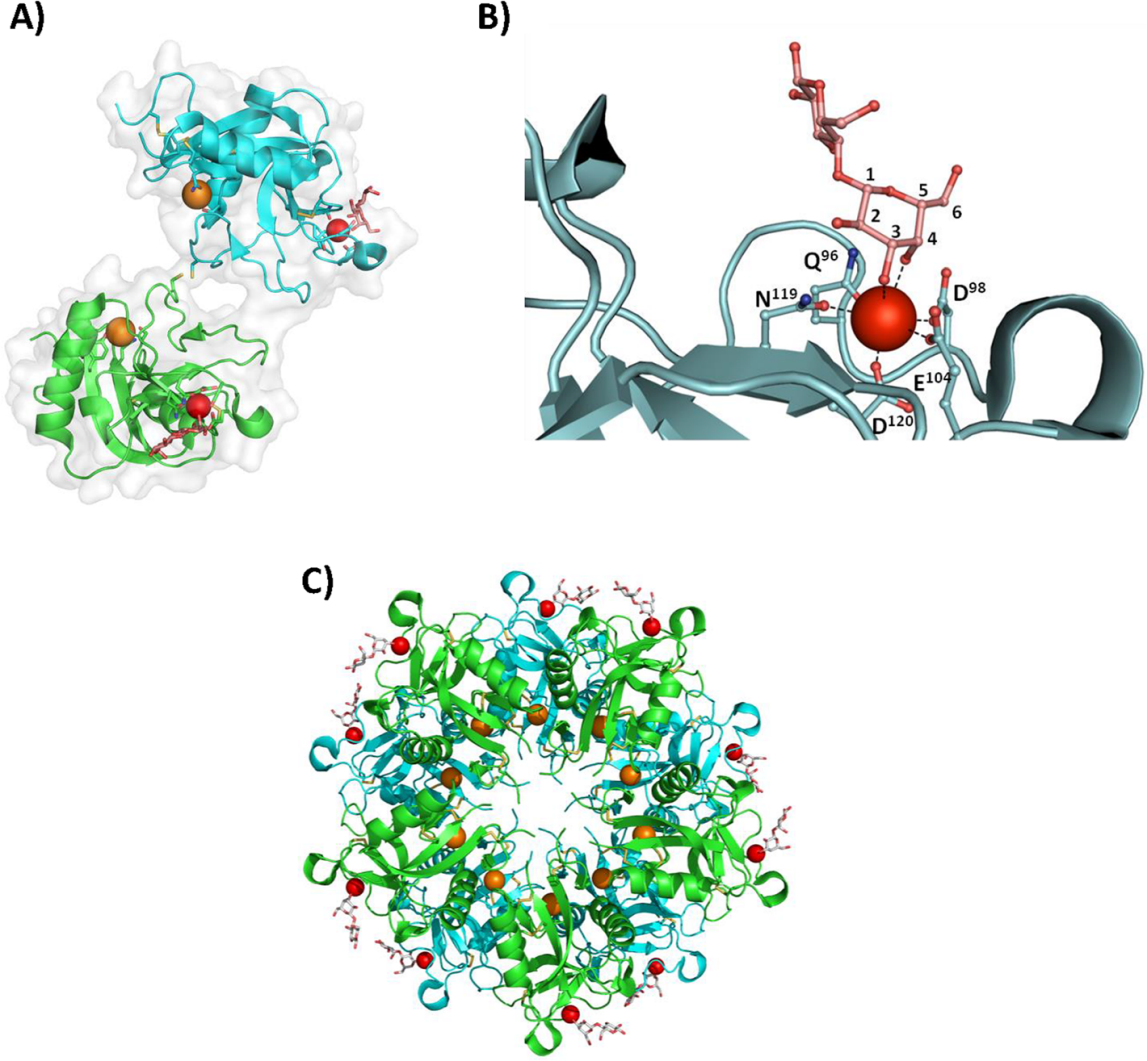
RSL tertiary structure and carbohydrate binding site. RSL structure as described elsewhere [22]. **a** A cartoon representation of the RSL dimer complexed with lactose. Cysteine side and inter chains are shown in yellow, calcium and sodium ions are represented as red and orange circles, respectively, and lactose is shown in stick representation colored in red. **b** Cartoon representation of the carbohydrate-binding site interacting with lactose. **c** Oligomerization state with the decamer viewed down the pseudo-5-fold rotation axis. The 3D structure illustration was performed using the software Pymol (The PyMOL Molecular Graphics System, Version 1.5.0.4 Schrödinger, LLC.) from RSL entry [PDB: 1jzn]

Secondary structural content analysis by circular dichroism was assessed for certain SVgalLs. The *Bothrops leucurus* Lectin (BleucL) and LmL contained mainly β structures (68 and 78 % respectively) with only 1 % α structures, thus being classified as a β class protein, while BJcuL possesses 18.8 % α-helix and 32.2 % β-sheet, and is classified as a α + β class protein [20, 30]. Walker et al. [22] determined that RSL structure is comprised of eight β-sheets and two α-helixes (Fig. 2a). As observed in Fig. 1, RSL amino acid residues involved in secondary structure are composed of highly conserved residues within other SVgalLs. This secondary structure conformation is supported by intermolecular interactions involving intra-monomeric amino acid segments. Residues H^99^ and F^105^ in conjunction with D^72^ from RSL, and conserved in the other lectins, appear to be involved in the stabilization of the loop between β-sheet 4 and β5, the CRD site, and β7 (Fig. 1) [22]. As to the dimeric form, in addition to cystine interaction of intermonomeric, electrostatic interactions between RSL monomers interface are maintained by salt bridges and hydrogen bonds [22].

In general, C-type galactoside-binding lectins CRD motifs are represented by three amino acid residues, Gln-Pro-Asp, which are considered to be determinants of their galactose specificities, where this carbohydrate interaction is mediated by calcium ions [31]. As shown in Fig. 1, this motif is fully conserved within all SVgalLs with primary structure determined, indicating its common carbohydrate recognition activity. As observed in the RSL structure complexed with lactose in Fig. 2b, the CRD is responsible for coordinating the calcium ion by interacting with the residues Q^96^, D^98^, E^104^, N^119^ and D^120^. The interaction with galactose residue from lactose (a disaccharide composed of galatose-glucose) occurs by coordination of galactose hydroxyl groups at position 3 and 4 of the hexose ring with the calcium ion (Fig. 2b) and by direct hydrogen bonds to residues Q^96^, D^98^, E^104^ and N^119^. Furthermore, the residue Q^121^ is involved in water-mediated hydrogen bond coordination of the 2-hydroxyl group of the galactose moiety, while residue Y^100^ also interacts with the galactose ring [22].

The aspects involving the galactose binding specificity of lectins are related to the hydrogen-bond interaction of residues Q^96^ and D^98^ with the galactose 3 and 4-hydroxyl groups, as observed in RSL [22], differently from mannose binding protein in which CRD is composed of an EPN motif (glutamic acid, proline and asparagine), whereas interaction with mannose 3-OH involves coordination of the calcium ion and simultaneous hydrogen bonding to residues E and N [32]. Aside from its CRD responsible for galactose binding activity mediated by calcium ion, RSL shows an alternative ion binding site that coordinates a sodium ion by residues Y^15^, S^42^, and Q^132^ and a water molecule, (Fig. 2a) [22]. This ion binding site is thought to be important in stabilizing RSL conformation [22], where the presence of these amino acids is fully conserved in the other SVgalLs and might also have an effect on these lectins (Fig. 1).

It has been reported that SVgalLs are capable of forming high-order oligomers [22, 33]. The X-ray crystallography structure of RSL reveals a decameric protein composed of five disulfide-linked dimers (ten monomers in total) arranged as two pseudo-5-fold symmetric pentamers as shown in Fig. 2c. As in the case of RSL, evaluation of BJcuL quaternary structure using different computational methods and biophysical experiments such as small-angle X-ray light scattering, revealed that the lectin, in solution, is a globular protein with molecular mass of 147.5 kDa with indications that BJcuL also forms an oligomerized decameric structure [33].

The decameric complex of RSL is composed of five dimers (Cys^86^–Cys^86^ disulfide-linked monomers) arranged as two pentamers (Fig. 2c), where its oligomeric structure is mainly maintained by four salt bridges and apolar interactions [22]. The fact that the RSL decameric structure presents ten carbohydrate-binding sites located on the edge of the two pentamers strongly suggests that the lectin presents a multivalent capacity of ligand-binding activity, as shown by its ability to induce erythrocyte agglutination due to cross-linking with the opposing cell surface. The ability to mediate multivalent interactions with different biological glycoconjugates have been described for others SVgalLs, such as galatrox, from *Bothrops atrox* snake venom, which induces a pro-inflammatory activity through its interaction with galactose-bearing glycoconjugates on the surface of neutrophils and macrophages and extracellular matrix (ECM) proteins [34].

### Glycan specificity

Given the effect of lectins on biological functions, understanding the molecular basis of carbohydrate recognition pattern comprises a relevant issue in lectinomics [35, 36]. So far, with the exception of the mannose-binding lectin isolated from *Oxyuranus scutellatus* crude venom [37], most snake venom glycan-binding lectins present the ability to interact specifically with sugars via terminal galactoside residues [6]. Interestingly, this similarity of galactose recognition patterns is reflected in several aspects among SVgalLs, such as the purification procedure which, for most snake lectins, is performed by single step liquid chromatography using affinity resins with a matrix composed of lactose- or galactose-based components [8–21, 38–41].

The major reported efforts to evaluate the carbohydrate binding specificity of SVgalLs consisted of assessing the capacity of sugars to inhibit erythrocyte agglutination activity. The results obtained for each SVgalLs assessed shows that glycans with terminal galactoside residue presented the most inhibitory efficiency. Although a similar galactose recognition pattern among these lectins, possibly associated with the structural aspects related to the CRD motif, variations in galactoside residues such as substitution of secondary disaccharide linkage or introduction of substituent e.g. methyl or amine groups on terminal galactoside residue intensifies or decreases lectin recognition specificity (Table 1).

**Table 1:** Carbohydrate inhibition specificity of SVgalLs

SVgalL	Snake species	Terminal galactoside carbohydrates (inhibition order)
ApL	*Akgistrodon piscivorus piscivorus* [15]	D-galactose > Lactose > N-acetyl-D-galactosamine
Thrombolectin	*Bothrops atrox* [9]	Thiodigalactoside > Lactose > Methyl β-D-galactopyranoside > Methyl α-D-galactopyranoside > D-galactose
BiL	*Bothrops insularis* [16]	Thiodigalactoside > Lactose > D-galactose > N-acetyl-D-galactosamine
BjL	*Bothrops jararaca* [12]	Thiodigalactoside > Lactose = N-acetyl-lactosamine > Methyl β-D-galactoside = D-galactose > Methyl α-D-galactoside > N-acetyl-D-galactosamine
CML	*Akgistrodon piscivorus leukostoma* [9]	Thiodigalactoside > Lactose > Methyl β-D-galactopyranoside > D-galactose >
		Methyl α-D-galactopyranoside
BJcuL	*Bothrops jararacussu* [14]	Lactose > D-galactose > D-galactosamine
RSL	*Crotalus atrox* [9]	Thiodigalactoside > Lactose > Methyl β-D-galactopyranoside = D-galactose >
		Methyl α-D-galactopyranoside
BleucL	*Bothrops leucurus* [20]	D-galactose > Lactose > N-acetyl-D-glucosamine > asialofetuin
BpaL	*Bothrops pauloensis* [21]	Lactose > D-galactose > N-acetyl-D-galactosamine
BpL	*Bothrops pirajai* [17]	Lactose > D-galactose
CuHL	*Akgistrodon contortrix contortrix* [9]	Thiodigalactoside > Methyl β-D-galactopyranoside > Lactose > Methyl α-D-galactopyranoside > D-galactose
CrL	*Crotalus ruber* [18]	Thiodigalactoside > N-acetyl-D-galactosamine = Lactulose > Lactose =
		Methyl β-D-galactoside > D-galactose
LmL	*Lachesis muta stenophyrs* [10]	Thiodigalactoside > Lactose > Methyl β D-galactopyranoside > D-galactose >
		Methyl α D-galactopyranoside > Galactosamine
ToL	*Trimeresurus okinavensis* [27]	Thiodigalactoside = Lactose
JML	*Dendroaspis jamesonii* [10]	Thiodigalactoside = Lactose > Methyl α D-galactoside > Metyl β D-galactoside > D-galactose > Galactosamine

**Fig. 3:**
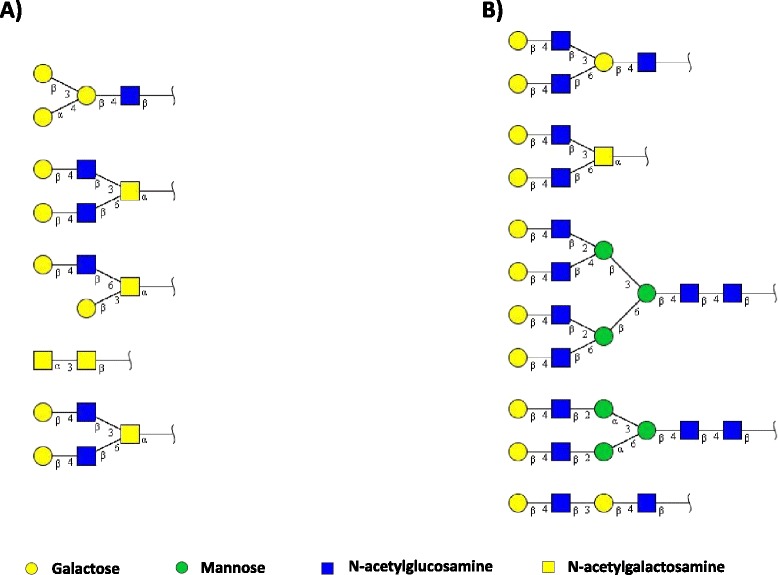
Glycan-recognition specificity. Top five glycan structures with highest binding recognition by the SVgalLs (**a**) RSL and (**b**) galatrox assessed by glycan microarray as described elsewhere [34, 42]

Lately, the advances in protein-carbohydrate recognition methods have included the introduction of new techniques, thus opening doors to the improvement of protein-sugar specificity using a variety of glycans that mimic biological sources of glycoconjugates [36]. More refined data on the investigation of carbohydrate-SVgalL interaction were obtained for RSL [42] and Galatrox [34] by performing the glycan microarray at the Consortium for Functional Glycomics (Protein-Glycan Interaction Core). The technique comprises a solid phase system screening to evaluate the recognition of proteins (such as lectins) in a wide range of immobilized glycans structures [43]. Both SVgalLs assessed presented a similar pattern of carbohydrate recognition to N-acetyllactosamine glycans, composed of terminal galactose (Gal) residue bonded to N-acetyl-glucosamine (GlcNAc) at the non-reducing end of the glycans (Fig. 3a and b). This finding is consistent with the results on hemagglutination inhibition (Table 1). Young et al. [42] observed that among over 264 glycan structures, RSL presented binding affinity preferentially to glycans with terminal Gal(α or β) or GalNAc(α) residues bonded to GlcNAc (Fig. 3a). As for galatrox [34], the lectin presented a significant specific preference for terminal Gal(β) bonded to GlcNAc glycans among over 406 structures analyzed (Fig. 3b). Additionally, the modification of terminal lactosamine structures, such as insertion of fucose or sialic acid on terminal Gal residues or substitution of the secondary monosaccharide from GlcNAc, disfavors the recognition intensity of both SVgalLs [34, 42]. These results have been highly constructive for elucidating the carbohydrate recognition pattern of these SVgalLs to different biological glycans, and have facilitated the elucidation of molecular mechanisms of these lectins in the biological events later described in this work.

### Biological functions

Glycans are found in living systems as free oligosaccharides or as glycoconjugates present on the cell surface and ECM, and are involved in a broad spectrum of biological functions including the signaling, recognition and adhesion of molecules [1, 36, 44]. The recognition of specific glycans by lectins represents a key event in a variety of biological phenomena involving cell-cell and cell-ECM component interactions, as observed in cell migration, apoptosis, immunomodulation and inflammation, host pathogen interactions and mitogenic induction [1, 45].

The SVgalLs comprise a group of exogenous glycan binding proteins (GBP) that have been reported to mediate several biological functions associated with snake envenomation pathophysiology and other biological properties [8, 11, 13, 34, 46–49]. The present section will approach functional features of SVgalLs and the association of their carbohydrate recognition specificity in these activities.

#### Mitogenic activity

In addition to hemagglutination activity, the ability of lectins to stimulate lymphocytes to undergo mitosis has been described from the early 1960s [50]. Since then, several other lectins from different sources have been reported to induce cell mitogenisis through recognition of sugars on the cell surface leading to signal transmission [3].

Subsequent to the early reports on biological characterization of SVgalLs, authors have assessed their ability to induce mitogenic activity. Mastro et al. [46], in a study working with five different SVgalLs, have shown that RSL, cottonmouth lectin (CML—isolated from *Akgistrodon piscivorus leukostoma* venom) and *Dendroaspis jamesonii* lectin (Jameson’s mamba lectin—JML) were capable of stimulating lymphocytes to undergo DNA synthesis, unlike thrombolectin and copperhead lectin (CuHL—isolated from *Akgistrodon contortrix contortrix* venom). The authors also showed that lymphocyte mitogenic activity of CML was preferentially induced in T-cells rather than B-cells, and was mediated by its carbohydrate binding properties. Moreover, CML macrophages stimulation increased lymphocyte proliferation presumably by involving interleukin-1 production from macrophages [46].

Other SVgalLs have also been assessed for their mitogenic activity, in which ToL was described as inducing the proliferation of human peripheral lymphocytes, mouse macrophages and bovine arterial endothelial cells [27]. On the other hand, *Bothrops godmani* Lectin (BgL) induced no mitogenic activity in human peripheral blood mononuclear cells [11]. The variation observed in the mitogenic activity among SVgalLs may be associated with the carbohydrate-binding specificity of these lectins, associated with a glycosylation pattern of cell surface receptors that are involved in this phenomenon [46].

#### Platelet aggregation

Investigations into the involvement of SVgalLs in the hemostasis system evidenced that these lectins act exclusively on platelet functions, as shown by the platelet aggregation agonists BmLec [24], RSL [51], *Agkistrodon piscivorus leukostoma* lectin (AplLec [51]), ToL [27] and LmL [51]. The proposed mechanism of platelet activation was based on the report of Ogilvie et al. [51], which showed that LmL recognizes glycans on the platelet surface and induces platelet aggregation mediated by glycoprotein IIb/IIIa signaling. Therefore, considering the binding specificity of SVgalLs, these results also indicate the presence of terminal galactoside residues on platelet surface glycoconjugates, where the recognition by the lectins seems to initiate the platelet stimulation and possibly induce blood coagulation disorders associated with snakebites.

On the other hand, several other lectins were not capable of inducing or inhibiting platelet aggregation, namely CuHL [51], galatrox [19], BjL [12], BJcuL [41], thrombolectin [51] and CrL [18]. This functional diversity involving platelet function may be associated with differences in the carbohydrate recognition specificity among SVgalLs [51].

#### Inflammation

Several endogenous and exogenous GBPs have been described as playing a pivotal role by regulating the participation of a wide variety of events during inflammation [52–56]. The participation of SVgalLs in inflammatory response has been assessed using different animal models and *in vitro* approaches in order to evaluate leukocyte involvement and recognition of ECM glycoconjugates in the inflammatory response.

Lomonte et al. [11] evidenced that BgL was capable of inducing moderate acute phase mouse paw edema that was inhibited by the antihistamine drug cyproheptadine. A similar pattern of acute mouse paw edema was observed in BJcuL, an effect that was associated with an increase in local vascular permeability [41]. Considering that galatrox and LmL were not capable of inducing mouse paw edema [19, 30, 57], the above results indicate that SVgalLs-induced paw edema might be associated with the capacity of the lectins to stimulate resident mast cell degranulation and the release of histamine and/or serotonin in order to trigger local inflammatory response.

In addition to paw edema, investigations concerning different sites of SVgalLs-induced inflammation and the involvement of leukocytes and ECM glycoproteins have been assessed. Elifio-Esposito et al. [58] demonstrated that BJcuL induced an increase in rolling and adherence of leukocytes observed in cremaster muscle microvessels of mice with topical administration of the lectin. Additionally, the authors also demonstrated that the lectin is capable of binding to the glycoproteins’ fibronectin and vitronectin, assuming that the extravasation of peripheral blood leukocytes into the inflammatory site involves cellular adhesion to ECM proteins mediated by BJcuL [58]. In order to confirm this evidence and elucidate the involvement of neutrophils in BJcuL-induced inflammation, a continuous study by Elifio-Esposito et al. [49] was performed by applying *in vitro* approaches. The authors observed that BJcuL not only recognizes glycoligands on the neutrophil cell surface, but also promotes polarization and migration, and enhances adhesion to the ECM components fibronectin and matrigel. The lectin also induced neutrophil functional activation by increasing phagocytosis and superoxide production [49].

Similar findings were also reported by Sartim et al. [34], who evaluated the pro-inflammatory response of galatrox. Although the lectin was not capable of inducing paw edema [19], in mice it was capable of promoting acute neutrophil migration into the peritoneal cavity and the release of cytokines IL-1α and IL-6. Moreover, galatrox was capable of recognizing neutrophil cell membrane glycoconjugates, of interacting with the polylactosamine ECM component laminin, and also inducing neutrophil chemotaxis *in vitro* [34]. Interestingly, the possible involvement of resident cells on galatrox-induced inflammation was assumed due to the ability of the lectin to stimulate macrophages *in vitro* to produce the pro-inflammatory mediators IL-6 and TNF-α, and keratinocyte-derived chemokine by interacting and triggering toll-like receptor 4 signaling in an MyD88-dependent pathway [34].

The above results show that the inflammatory-induced mechanism of the mentioned SVgalLs is characterized by neutrophil influx to the inflammation site promoted by direct lectin-ECM interaction chemoattractive role and/or through stimulation of resident macrophages or mast cells in order to express soluble mediators responsible for amplifying the inflammatory response. An exceeding inflammatory process induced by these lectins, and others venom components, may be associated with snake envenomation pathophysiology related to tissue damage and organ injury [59, 60].

#### Renal effects

During snake envenomation, renal disorders represents an important clinical manifestation where the kidney represents an organ vulnerable to venom components due to its high degree of vascularization. Multiple factors including hemodynamic changes, inflammatory reactions and nephrotoxic effects of toxins are responsible for several manifestations such as proteinuria, hematuria and renal failure [61]. Studies involving the evaluation of renal disorders induced by SVgalLs in isolated rat kidneys have been reported. The lectins BiL, BpirL and BmLec were capable of inducing alterations in renal functional parameters such as perfusion pressure, renal vascular resistance, urinary flow, glomerular filtration rate and tubular ion transport [17, 24, 48]. Moreover, indomethacin, an anti-inflammatory drug that inhibits lipidic mediator prostaglandin synthesis, completely inhibited all evaluated parameters of renal alterations induced by BmLec [24]. Additionally, histological analysis of kidneys from animals that received BiL suggested direct injury to glomerular and tubular renal cells [48]. Therefore, a rational proposal on the mechanism of renal alterations suggested by the authors indicates a direct cytotoxicity induced by the lectins at the glomerular level and/or the involvement of systemic pro-inflammatory mediators such as prostaglandins, suggesting their involvement in envenomation-induced renal disorders [17, 24, 48].

#### Other biological functions

As mentioned above, SVgalLs are able to recognize and interact with several biological glycoconjugates in order to induce their functional activity, being associated with snakebite-related pathologies. However, given the advance of emerging techniques applied to determine the carbohydrate composition of glycoconjugates in association with the large amount of data on lectin-glycan recognition, the investigation of novel applications of these snake venom GBPs has been largely completed [1, 36, 43].

Ryanodine receptors are calcium-release channels expressed in several human cells such as those present in sarcoplasmatic reticulum (SR) from skeletal muscle, and are associated with muscle contraction [62]. A wide variety of compounds from diverse natural sources have been assessed as molecular approaches to elucidate the molecular mechanism of calcium-release involving ryanodine receptors. Among these chemical probes, the snake venom lectins ToL and BaL were reported to trigger calcium release specifically from a heavy fraction of skeletal muscle SR (HSR) by interacting with HSR ryanodine receptors, where the binding sites by which both SVgalLs interact appeared to be different from those sites able to recognize the well known agonists caffeine, ryanodine and myotoxin alpha, isolated from *Crotalus viridis viridis* venom [39, 47]. These findings indicate both lectins as possible novel pharmacological tools for application in functional studies of calcium release from HSR in skeletal muscle.

Considering that during the infection process pathogen adhesion to the host cell involves protein-glycan interactions [63, 64], glycoconjugates from the pathogen cell surface represents a potential target for SVgalLs. Castanheira et al. [21] showed that BpalL, a lectin isolated from *Bothrops pauloensis* venom, induced agglutination but not toxicity of promastigote forms of *Leishmania amazonesis* through interaction with galactoside glycoconjugates on the parasite membrane. The effects of BpalL on bacteria were also evaluated, where the lectin inhibited gram-positive *Staphylococcus aureus* growth, but not gram-negative *E.coli* [21]. Similar findings were also obtained for BleucL, isolated from *Bothrops leucurus* venom, which promoted antibacterial activity against gram-positive bacteria *Bacillus subtilis*, *Staphylococcus aureus* and *Enterococcus faecalis*, but not against gram-negative *E. coli* or *Klebsiella pneumonia* [20]. The authors suggested that the difference in susceptibility is associated with the interaction of BpalL and BleucL with peptidoglycan present in the gram-positive bacterium cell wall, while the lectins may not reach the same targets on Gram-negative due to the outer cell wall [20, 21]. In contrast, BmLec, isolated form *Bothrops moojeni* venom, induced bactericidal activity against gram-negative *Xanthomonas axonopodis pv. passiflorae*, where ultrastructural analysis showed that the lectin binds to the membrane resulting in the formation of the bacterial aggregates, and also the development of large vesicles in the bacterial cell membrane that lead to membrane rupture [24]. This contrast in relation to the effects to gram-negative bacteria among the mentioned SVgalLs may be associated with the variation of carbohydrate binding specificity of these lectins to outer cell wall glycoconjugates or even to morphological singularities among bacteria [65].

Genetic alterations in malignant tumor cells are associated with changes in their glycosylation patterns when compared to normal cells, which include an increased expression of unusual terminal carbohydrate sequences [66, 67]. Considering that this novel pattern of surface glycoconjugates might develop malignant cells into novel targets for lectins, a progressive number of investigations on the involvement of SVgalLs in cancer cells have been launched. The evaluation of SVgalLs-induced cytotoxicity *in vitro* became the most common effort reported where, as shown in Table 2, the lectins assessed were capable of inducing toxicity against a wide variety of tumor cell lines through recognition of membrane glycoconjugates. Most cell death was reported to be induced by apoptotic signals as shown for BJcuL against the MKN45, AGS and HT29 cell lines [68, 69], and BleucL against the K562 cell line [70]. Interestingly, apoptosis represents an essential mechanism by which several chemotherapeutic agents act [71], indicating a potential applicability of these lectins. As to intracellular signaling mechanisms of cell death, Damasio et al. [69] observed that BJcuL triggers a cascade of kinases via the activation of tumor necrosis factor-related apoptosis-inducing ligand receptors on HT29 cell line, involving expression of Fas-associated death domain, caspase-8 and Bax proteins, and leading to cell apoptosis.

**Table 2:** SVgalL tumoral cell lines citotoxicity

SVgalL	Snake venom specie (reference)	Tumorigenic cell line
Galatrox	*Bothrops atrox* [19]	HL-60 (human promyelocytic leukemia)
BJcuL	*Bothrops jararacussu* [68, 69, 74]	MDA-MB- 435 (human breast carcinoma); OVCAR-5 (ovarian carcinoma); U87 and A-172 (glioblastoma); MKN45 and AGS (human gastric carcinoma); HT29 (human colon adenocarcinoma)
BleucL	*Bothrops leucurus* [70, 72]	K562 (human chronic myelocytic leukemia); NCI-H292 (human lung mucoepidermoid carcinoma); Hep-2 (human larynx epidermoid carcinoma); B16-F10 (melanoma)

Moreover, the authors also observed that the lectin increases the permeability of the external mitochondrial membrane and induces respiration decrease [69]. Similar findings on mitochondrial dysfunction were also observed for BleucL toxicity toward the B16-F10 cell line, which induced the increase of calcium concentrations in cytosol and mitochondrial superoxide generation, and favored mitochondrial permeability transition pore opening, leading to cell death by necrosis [72]. Despite its cytotoxicity toward tumor cell lines, BleucL presented no changes in viability of the nontumorigenic HaCaT cells derived from human keratinocytes, indicating a selective death-induced activity towards cancer cells [70, 72]. Aside from cytotoxicity, SVgalLs presented other features involving antitumor activity as evidenced by BJcuL which was capable of inhibiting adhesion of HT29, MKN45 and AGS cell lines to ECM components within matrigel, indicating an important mechanism to annul the attachment of cancer cells to tissue [68, 69]. The abovementioned findings suggest these lectins as potential anticancer agents owing to their abilities to recognize, kill and suppress common features of tumor cell lines during cancer progression.

## Conclusion

During the past 35 years, scientists have shown that snake venoms represent an important source of galactoside-binding lectins that are involved in diverse features of snake biology. The specificity towards terminal galactose residues confers to these molecules the ability to recognize a wide variety of glycoconjugates, and act as an important tool in glycan-codified functions. This capacity corroborates these lectins as potential approaches applied in basic research, clinical diagnosis and pharmacological therapy. Therefore, future studies on SVgalLs become crucial not only for the elucidation of their involvement in snake envenomation pathophysiology, but also for the development of biotechnological tools.
